# Natal origin of the invasive biosecurity pest, brown marmorated stink bug (*Halyomorpha halys*: Penatomidae), determined by dual‐element stable isotope‐ratio mass spectrometry

**DOI:** 10.1002/ps.5659

**Published:** 2019-12-09

**Authors:** Peter W Holder, Robert J Van Hale, Russell Frew, Sherly George, Karen F Armstrong

**Affiliations:** ^1^ Bio‐Protection Research Centre Lincoln University Christchurch New Zealand; ^2^ Department of Chemistry University of Otago Dunedin New Zealand; ^3^ Plant Health & Environment Laboratory Ministry for Primary Industries Auckland New Zealand

**Keywords:** brown marmorated stink bug, natal‐origin, stable‐isotope‐analysis, biosecurity, pest management

## Abstract

**BACKGROUND:**

Post‐border detection of a single brown marmorated stink bug (BMSB) in New Zealand warranted a biosecurity response, the nature of which would be influenced by its status as part of an established population or as a new arrival. Stable isotope analysis has the potential to determine natal origins, but is difficult to achieve for samples as small as a single insect. Here an analytical modification to measure small samples was successfully trialled as a means to supply evidence as to the local or exotic natal origin of the intercepted BMSB specimen.

**RESULTS:**

Sufficient analytical sensitivity was achieved using a modified isotope ratio mass spectrometry method, involving thermolysis and carbon monoxide cryofocusing, to enable the simultaneous analysis of δ^2^H and δ^18^O from wings of the post‐border BMSB sample. The values were much lower than those of the New Zealand green vegetable bug, used as a local reference. However, they fell within the range of those for BMSB of Northern Hemisphere origin intercepted at the New Zealand border over the same time period, specifically overlapping with the USA and Italy, but not China.

**CONCLUSION:**

The isotope signature of the post‐border detected BMSB suggested a significantly cooler climate than the North Island of New Zealand, indicating that it was a new arrival and did not represent an established population. © 2019 The Authors. *Pest Management Science* published by John Wiley & Sons Ltd on behalf of Society of Chemical Industry.

## Graphical abstract

Provenance determination of pest insects can improve biosecurity and pest‐management outcomes. Here, a brown marmorated stink bug incursion of unknown origin is investigated using δ^2^H and δ^18^O, enabled by a modified analytical method.

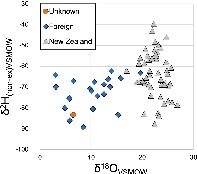

## 1 INTRODUCTION

Early detection of exotic plant pests improves the likelihood of their successful eradication.^1^, ^2^ This is the most effective way to protect the economic returns of plant‐based industries and plants of conservation value, as well as avoid the monetary,^3^ environmental^4^ and social^5^ consequences of long‐term pest management. Heightened surveillance systems aim to achieve early detection for priority pests.^6^ In such systems, detection may be of first arrivals or members of a recently established population. However, these two chronologically close scenarios require different biosecurity responses with very different resource investments. Compared to first arrivals, where biosecurity actions may cost from tens of thousands to a few hundred thousand dollars (NZ$), the financial impact of response to establishment can run into millions of dollars to delimit and eradicate the pest, and as a result of trade disruption (e.g.).^7^ A key question at the time of a detection is therefore ‘Does this represent an established population or not?’

The brown marmorated stink bug (BMSB) is one such priority, highly invasive, pest. Beyond its native range it is highly damaging to a vast range of plants and is a significant nuisance pest in urban areas.^8^ Originally from East Asia, it spread to North America in the 1990s, Europe in 2007 and very recently to South America in 2017 (confined to Chile).^8^ In New Zealand (NZL) BMSB remains amongst the top high‐risk pests not yet present there,^9^ with frequent risk of introduction^10^ and potentially severe impacts.^11^ Hence, when a single BMSB specimen was detected post‐border in NZL, the cautious view was taken that it may represent an established population. Even though the circumstances suggested the specimen may have hitchhiked (as an adult insect) with newly arrived tourists, there was no evidence, or existing mechanism by which to establish this evidence, that it was not part of an established population. The opportunity was therefore taken to develop a method to determine if the specimen had either local (established) or non‐local (not established) origins by considering its natural abundance stable isotope composition.

Stable isotopes are intrinsic to all matter, hence isotope signatures are incorporated into the tissues of organisms through their diet, they are not inherited. Therefore, unlike DNA, which is used to determine historical or inter‐generational origins by way of their inherited nature,^12^ the isotope composition of metabolically fixed tissues reflects the place in which the organism developed.^13^, ^14^ The value of this relationship to indicate insect natal origins has been demonstrated in an ecological context for many years (e.g.),^15^, ^16^, ^17^, ^18^ but the potential for biosecurity has only recently been recognized.^19^, ^20^, ^21^, ^22^

Hydrogen and oxygen stable isotope markers were considered here. These are composed of the isotope ratios within each element and are expressed as δ^2^H and δ^18^O, where delta (δ) is the isotopic composition of the sample relative to the calibration standard, Vienna Standard Mean Ocean Water (VSMOW) (see).^23^ As δ^2^H and δ^18^O are directly related to temperature,^24^ these markers have strong potential to distinguish geographic origin according to latitude and altitude.^25^, ^26^ Additionally, as the risk pathways to NZL for BMSB are known to be almost exclusively from Europe, the USA, and South‐East Asia,^10^ we hypothesized that these markers would be particularly informative for this case, given the seasonally linked climatic differences between the Northern (not NZL established) versus Southern (NZL established) Hemisphere source options over the development period of the intercepted specimen. At the time of this incursion, BMSB had not been confirmed as present in Chile. For provenance assignment in ecology, δ^2^H has been the most commonly used isotope marker, largely due to analytical accessibility. δ^18^O has been less commonly used due to analytical difficulties,^27^ especially with small samples (discussed below), plus assignment of provenance is potentially complicated due to animals sourcing O from air as well as water and food.^28^ However, provenance resolution can be improved by considering δ^2^H and δ^18^O together, as they serve as semi‐independent markers due to differences in evaporative response.^27^, ^29^, ^30^ Additionally and importantly, data derived from separate isotope systems also permits the use of more powerful, multivariate statistical analyses.^31^

Our choice of markers was also restricted by the biosecurity circumstance. First, the urgency required of a response, and second, the amount of tissue available in a single insect sample is less than required for most isotope analyses. Both factors preclude the preferred use of a more extensive set of markers.^20^ The constraint of mass is also exacerbated by the need to use only the insect flight wings. The elements comprising the adult wing accumulate during the non‐dispersing nymphal life‐stage(s). In contrast to the soft tissues and other parts of the insect exoskeleton, the flight wings undergo minimal cellular turnover during the adult live stage. Thus the isotope ratio of that tissue is expected to be fixed for the adult life stage^32^ and so represents the place of natal origin, rather than the more recent place of any adult feeding. Unfortunately, BMSB flight wings are on average only 105 μg (±28 μg 1 SD, *N* = 50) and although δ^2^H measurement is routinely possible from such samples, this mass is ∼0.6 of the minimum required for conventional solid bulk sample δ^18^O analysis. Hitherto measurement of δ^18^O in small insect samples has been achieved by pooling insects,^33^ but this is not possible in single insect incursions, which is predominantly the case in the biosecurity context, and so ongoing method development has been required for small sample, simultaneous δ^2^H and δ^18^O assay.

The primary aim here was to determine the likely origin of the BMSB intercepted inside New Zealand borders as local or exotic. This involved trialling a method incorporating cryofocusing of carbon monoxide (CO) from a thermal conversion elemental analyser (TC/EA) to address the aforementioned limitations with conventional δ^18^O analysis. This test was able to contribute evidence that the specimen intercepted inside New Zealand borders had just arrived in New Zealand and was not from a locally breeding population.

## 2 METHOD

### 2.1 Sample collection and processing

A single unmated female BMSB was collected alive in Whitianga, New Zealand, from the floor of a holiday apartment in February 2017. This represented a biosecurity incursion of unknown origin (New Zealand Ministry of Primary Industries reference: T17‐490^10^) and is subsequently referred to as ‘UnKSB’ (‘Unknown stink bug’).

Non‐NZL reference material was collated from recent at‐border interceptions of BMSB. These were from separate interceptions made during routine border biosecurity inspections over the 3 months prior, i.e. the same season for arrival in NZL as UnKSB, if it was of non‐NZL origin (Table 1). As BMSB is not present in NZL, NZL reference material required the use of a closely related pentatomid, the green vege bug (GVB) (*Nezara viridula*) as a species‐surrogate. These had been collected from various NZL regions through 2014–2016 (Table 1). Only samples collected during the time of year that corresponded with the development time of UnKSB (see)^34^, ^35^ were used.

**Table 1:** Collection information for UnKSB (T17‐490), intercepted, non‐NZL BMSB reference samples, GVB sampled in New Zealand used to provide a species‐surrogate NZL reference data set, and BMSB and GVB sampled in California, USA that were used to estimate the suitability of GVB as a species‐surrogate for BMSB

Species	Country/region of origin	Collection details/found on	No. of specimens analysed	Collection date
BMSB	Unknown	Whitianga, NZL, Holiday apartment floor	1	20 February 2017
*Intercepted, non‐NZL BMSB reference samples*				
BMSB	USA	Luggage	1	17 February 2017
BMSB	USA	Vehicle	1	13 February 2017
BMSB	Italy	Foodstuffs	2	13 February 2017
BMSB	USA	Sea container	5	9 February 2017
BMSB	Italy	Box	1	27 January 2017
BMSB	Italy	Car parts	1	27 January 2017
BMSB	Taiwan	Tiles	1	24 January 2017
BMSB	China	Unknown	5	19 January 2017
BMSB	Italy	Steel iron equipment	5	11 January 2017
BMSB	Hungary	Personal effects	2	29 December 2016
*GVB sampled in New Zealand*				
GVB	Northland	Various vegetables spp.	27	November 2014–February 2015
GVB	Auckland	Various vegetables spp.	13	November 2014–February 2015
GVB	Bay of Plenty	*Phaseolus vulgaris* (green beans)	3	January 2015
GVB	Mid‐Canterbury	Various vegetables spp.	6	January 2015
*BMSB and GVB sampled in California, USA*				
BMSB	Sacramento, CA, USA	*Helianthus annus* (sunflower)	12	18 August 2015
GVB	Napa co., CA, USA	*Phaseolus vulgaris* (green beans)	14	1 October 2015

In addition, to determine if there is a species‐related difference in isotope expression (‘fractionation’) between BMSB and GVB, isotope data from specimens of both species collected at Californian (USA) sites in close geographic proximity were compared (Table 1).

All insect samples were killed by freezing and stored at −20 °C until pre‐analysis processing. Both flight wings were dissected from the adult beetles, washed three times with 2:1 chloroform: methanol solution to remove oils^36^, ^37^ then air dried for 12 h.

### 2.2 Stable isotope analysis

#### 2.2.1 *Sample and standard preparation*

Flight wings from individual insects were loosely crimped in 3 × 5 mm silver capsules (OEA Laboratories, Kelly Bray, Cornwall, United Kingdom). Target weights were ∼130 μg per sample, representing H and O masses of 8 μg and 30 μg, respectively. These samples were then equilibrated with laboratory air for 6 days, then dried at 60 °C under vacuum for 4 days. The international reference materials USGS‐42 and USGS‐43 (human hair) were used as sample matrix‐matched calibration and drift correction standards, and treated identically to the samples.^38^ USGS‐53 (Lake Shala Distilled Water) and IAEA‐CH7 (polyethylene foil) standards provided scale correction for raw δ^2^H, while USGS‐53 provided a three‐point calibration for raw δ^18^O with the two USGS human hair standards. Because of the range of sample weights, a USGS‐43 size series of 20–250 μg was measured with the samples to allow the calculation of mass dependence corrections, if required. The quality control results reported are from repeated measurements of 130 μg of USGS‐43 interspersed with the samples.

#### 2.2.2 *Dual δ^2^H and δ^18^O measurement*

Isotope measurements were conducted at University of Otago (Dunedin, NZL) using a Costech Zero‐Blank autosampler mounted on a Thermo Scientific ™ thermal conversion elemental analyser (TC/EA), coupled to a Thermo Scientific™ Delta V™ isotope ratio mass spectrometer (IRMS) in continuous flow mode. The reactor temperature was 1400 °C and the gas chromatograph (GC) 83 °C.

The existing method for δ^18^O measurement by TC/EA requires 80 μg of O.^39^ To accommodate less than this, as typical of single insect samples, an approach involving cryofocusing CO^40^ to improve instrumental signal for O was utilized. This was coupled with the peak jumping method described by Qi *et al*. and Coplen and Qi^41^, ^42^ for dual measurement of H and O isotope ratios. Following the passage of the H_2_ peak from the GC column to the IRMS, the N_2_ was switched to waste by a ‘heart cut’ valve and CO was passed to a trap consisting of ∼2 cm^3^ of 45–60 mesh 5 Å molecular sieves held at liquid nitrogen temperature. After 450 s the trapped gas was released to the mass spectrometer in a 10 mL min^−1^ helium carrier by allowing the trap to approach room temperature. The δ^18^O value was determined using the ^12^C^18^O/^12^C^16^O ratio.

### 2.3 Data analysis

Where there was sufficient material, the two flight wings of individual insects were measured separately. In these cases, the average value (of the two wings) is reported.

The validity of GVB as a surrogate species for BMSB was assessed by considering the Californian BMSB versus GVB δ^2^H and δ^18^O data combined (MANOVA, GENSTAT 18), as well as in separate ANOVAs.

A discriminant function analysis (GENSTAT 18) was used to assess the relationship between the UnKSB sample and the reference data. This initially involved performing a discriminant analysis of the NZL versus ‘Foreign’ (non‐NZL) reference samples using the δ^2^H and δ^18^O data simultaneously. The resultant linear discriminant function was then used to estimate the probability that UnKSB belonged to the NZL group of reference samples or the Foreign group.

## 3 RESULTS

### 3.1 Simultaneous measurement of δ^2^H and δ^18^O in single BMSB specimens using cryofocused CO

**Figure 1:**
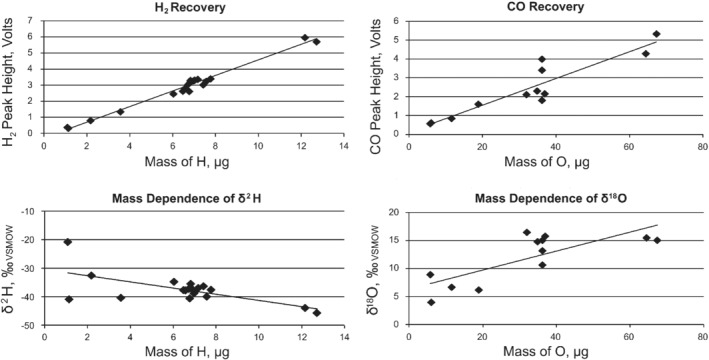
The effectiveness of the simultaneous δ^2^H and δ^18^O analytical method: signal intensity and isotope ratio mass dependency achieved with a size‐series of USGS‐43. Sample mass and H_2_ recovery was strongly co‐related, and mass dependence was acceptable for samples over 6.5 μg H. In contrast, there was variable CO recovery, which is likely to have contributed to the low precision parameters observed. Given this accuracy range, the linearity of δ^18^O measurement appears consistent down to ∼35 μg O.

The average measured δ^2^H value from repeated analysis of ∼130 μg USGS43 samples was within ∼1‰ of the internationally accepted value (Table 2), and precision was better than the laboratory long‐term average (±3‰, with standard method). Figure 1 indicates repeatable H_2_ recovery, and precision was consistent for samples containing more than ∼6.5 μg H. Thus, the method delivers acceptable δ^2^H analysis.

**Table 2:** Quality control parameters for δ^2^H and δ^18^O analyses achieved with cryofocused CO, using ∼130 ug samples of Standard USGS‐43 (*N* = 15)

Compound		δ^2^H‰ ± 1 SD	% H	δ^18^O‰ ± 1 SD	% O
USGS‐43	Accepted	−44.4 ± 2.0	6.1	14.11 ± 0.10	22.00
	Measured	−43.41 ± 1.8	6.10 ± 0.07	12.6 ± 3.5	19.6 ± 2.5

On the other hand, the cryofocus analyses yielded δ^18^O accuracy at ∼1.5‰ less than the accepted value (Table 2), and precision of ±3.5‰ (one standard deviation (SD)). Within this accuracy range, the δ^18^O measurement appears linear down to ∼35 μg O (representing ‘bulk’ sample weight of ∼160 μg). Although the analytical precision for δ^18^O measurement is poor, data generated using the method was used for the following comparisons as the differences observed between treatments (groups) was much larger than the potential analytical error.

### 3.2 Assessing GVB as a surrogate species for BMSB

A statistically non‐significant MANOVA effect was obtained for Californian GVB versus BMSB data, when using a 95% confidence level (*F* (2,23) = 2.99, *P* = 0.07, *perm. prob*. = 0.068, Wilk's Λ = 0.7938). Univariate assessment shows that the δ^2^H data for BMSB and GVB was virtually identical (*F* (1,24) = 0.04, *P* = 0.835), with mean values for the two species less than one permil different. In contrast, the δ^18^O data was different between the species (*F* (1,24) = 5.85, *P* = 0.024). However, the species means are only 1.3‰ different, potentially reflecting the difference in host plant and subtle differences in season and location of collection. Additionally, the range in values broadly overlaps for the different species (Table 3).

**Table 3:** δ^2^H and δ^18^O data from UnKSB (T17‐490), intercepted, non‐NZL BMSB samples, GVB sampled in New Zealand used to provide a species‐surrogate NZL reference data set, and BMSB and GVB sampled in California, USA that were used to estimate the suitability of GVB as a species‐surrogate for BMSB

Species	Country/region of origin	No. of specimens analysed	Mean δ^18^O (1 SD)	Mean δ^2^H (1 SD)
BMSB	Sacramento, CA, USA	12	25.972 (1.493)	−92.66 (12.27)
GVB	Napa co., CA, USA	14	24.594 (1.409)	−93.59 (10.42)
BMSB	Unknown	1	6.5^a^	−83.4^a,b^
BMSB	USA	5	8.86 (5.12)^a^	−78.02 (9.67)^a,b^
BMSB	Italy	8	7.75 (3.49)^a^	−74.34 (6.72)^a,b^
BMSB	Taiwan	1	9.8^a,b^	−70.68^a,b,c^
BMSB	China	5	14.02 (3.52)^b^	−67.17 (4.25)^b,c^
BMSB	Hungary	2	13.24 (3.62)^b^	−66.64 (1.42)^a,b,c^
GVB	Northland	27	23.3 (1.9)^e^	−63.4 (9.8)^c^
GVB	Auckland	13	21.5 (1.0)^c,d^	−58.6 (10.4)^c^
GVB	Bay of Plenty	3	19.1 (1.9)^c^	−56.6 (5.1)^c^
GVB	Mid‐Canterbury	6	23.4 (1.8)^d,e^	−78.4 (6.5)^a^

Superscript letters indicate Fisher's LSD ANOVA grouping for UnKSB versus origin reference samples (LSD = 5%).

### 3.3 Assessment of unknown BMSB sample provenance

The isotope measurements of UnKSB, along with NZL GVB and foreign BMSB (assigned on the circumstances of their detection) reference samples are summarized in Table 3.

**Figure 2:**
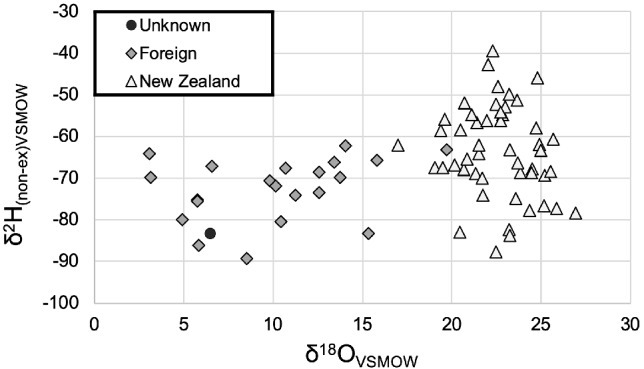
Natal origin assessment of a suspected BMSB biosecurity incursion by dual‐element stable isotope‐ratio mass spectrometry: δ^2^H and δ^18^O values (‰) for UnKSB together with reference samples composed of intercepted Foreign BMSB and NZL GVB specimens collected during the appropriate season. These data strongly indicate that UnKSB did not represent a New Zealand established population.

The Foreign and NZL reference data sets were clearly different, and most distinctly so with δ^18^O (Fig. 2). The linear discriminant function (LDA) generated from this reference data is shown in Table 4. The post‐hoc multi‐variate assessment, using this LDA, strongly assigned UnKSB to the Foreign group (Probability (Foreign) = 1.000 (squared distance 2.296); Probability (NZL) = 0.000 (squared distance 32.034).

**Table 4:** Linear discriminant function for the Foreign versus NZL reference data sets

	Foreign	NZL
Constant	−31.471	−48.492
δ^2^H	−0.706	−0.622
δ^18^O	1.153	2.543

The most informative univariate result arose from the δ^18^O data:

1. The δ^18^O value of UnKSB was 16.1‰ less than the NZL average value (i.e. 6.5‰ versus M = 22.6‰ (SD = 2.1‰), respectively) and was clearly highly significantly different (*F* (1,48) = 58.96, *P* < 0.000).
2. In contrast, UnKSB δ^18^O value was not significantly different from any of the Foreign groups individually (*F* (5,16) = 2.04, *P* = 0.127) nor significantly different from the foreign reference δ^18^O data overall (M = 10.1‰ (SD = 4.5‰); *F* (1,20) = 0.63, *P* = 0.438).

The δ^2^H data were less different between the groups, but were still informative. The overall δ^2^H NZL mean (M = −63.6‰, SD = 11.1‰) was 19.8‰ more positive than UnKSB and, although the δ^2^H value of UnKSB was not significantly different overall from NZL at α = 0.05 (*F* (1,48) = 3.15, *P* = 0.082), this was largely due to the large confidence interval (44.4‰) around UnKSB due to the sample size being *N* = 1. Of more direct geographical relevance, the UnKSB δ^2^H was significantly more negative than the δ^2^H data from the northern NZL regions (where Whitianga is located) individually, but not significantly different to Mid‐Canterbury, which is a cooler southern NZL region (*T* (1,6) = −0.48, *P* = 0.633, Table 3).

In contrast, the δ^2^H value of UnKSB was ∼9‰ lower than the average of the foreign reference samples (M = −72.6‰, SD = 7.6‰). The δ^2^H value of UnKSB was not significantly different from the foreign reference δ^2^H data overall (*F* (1,20) = 1.91, *P* = 0.182) nor was it significantly different from any of the Foreign groups (individually, *F* (5,16) = 2.11, *P* = 0.117).

## 4 DISCUSSION AND CONCLUSIONS

In this study we present the novel use of an existing technique in delimiting the country of origin after incursion of an invasive pest species. Specifically, we have demonstrated that stable isotope analytical methods can be modified to yield results with small insect samples, and that analytical sensitivity was sufficient to yield data that grouped an incursive pest with others of the same species from the country from which the pest potentially originated. This demonstrates that this technique can be a valuable tool in informing the origin of samples comprising small mass insect pests, and thus directing appropriate pest management decisions.

Knowing the establishment status of pest species intercepted in biosecurity surveillance systems is crucial to effectively manage the containment response. The potential for stable isotope markers to determine this status, in the absence of any alternative, is compelling. The data presented here strongly indicate that the UnKSB Post Border BMSB (T17‐490) did not originate from its point of collection in New Zealand, and thus did not represent a New Zealand established population. More negative δ^2^H values and lower δ^18^O are indicative of development in cooler environments.^25^, ^43^ Both the H and O isotope values of the UnKSB sample were lower than expected values from Northland, Auckland and Bay of Plenty regions for that time of the year (spring–summer), which strongly indicates the T17‐490 sample originated in a significantly cooler climate. Moreover, UnKSB δ^2^H and δ^18^O values were 5‰ and 16.9‰ less, respectively, than that of Mid‐Canterbury GVB. This suggests that the climate of origin of the UnKSB is likely to be a region at least as cool as the south of NZL, but probably even cooler, consistent with the Northern Hemisphere risk pathways.

Here we have assessed a semi‐miniaturized assay for improved analytical sensitivity for δ^18^O, coupled to simultaneous measurement of δ^2^H and δ^18^O. The analytical precision for δ^2^H was satisfactory, but below that normally acceptable for δ^18^O, possibly due to the poor CO recovery (Fig. 1). Nevertheless, the sensitivity to the technique was sufficient to provide evidence for the origin of the incursion in the urgent biosecurity case presented, given the very large differences between the reference groups. The spatial discrimination achieved in this study further corroborates the value of biogeochemical markers for elucidating the origin of invasive alien species, plus it expands our understanding of the expression of light element isotope markers in insects. Hitherto, there has been little research on stable isotopes as provenance markers in sap‐sucking insects (e.g.)^44^ and none on the family Pentatomidae. Of note is that the intra‐population δ^2^H variation was less than observed for other entomological isotope studies^21^, ^45^ and the intra‐country variation was generally consistent across the reference data‐set. Consequently, the BMSB from the different countries cluster relatively closely, including those samples intercepted on separate, unrelated shipments (i.e. the USA and Italian interceptions). Thus, although the observed difference in δ^2^H between countries is small, and the regional data ranges overlap, the overall results afford confidence in interpretation with respect to assignment of UnKSB. Furthermore, the BMSB and GVB specimens collected from contiguous USA locations give very similar place‐to‐insect isotope ratio expression (non‐significant MANOVA, and the relatively subtle difference in δ^18^O data between the species relative to all other comparisons). Accordingly, we consider that GVB is an adequate surrogate species for BMSB for the purposes of this project.

The positive contributions given above withstanding, there remain some uncertainties and challenges that need to be addressed before there can be widespread application of biogeochemical markers in entomological provenance determination. Perhaps foremost among these is the potential effect of insects feeding on different host plants.^46^, ^47^ Indeed, this needs to be considered in the present study, as BMSB is a polyphagous insect. Given that the host plant of the UnKSB BMSB sample was not known, some of the differences observed between the NZL GVB and UnKSB could be attributed to different host plants used by the different groups.^46^ However, the maximum host effect observed elsewhere in GVB δ^2^ *H* was 14‰,^48^ which is less than the average NZL GVB versus UnKSB difference of ∼20‰. For δ^18^ *O* the maximum GVB host effect was 3‰,^48^ significantly less than the NZL GVB versus UnKSB difference of at least 10‰. Thus, the difference between the NZL GVB and UnKSB is considered greater than any potential effect of polyphagy.

Relatedly, our understanding of the location (from soil and precipitation) to insect expression of biogeochemical markers needs to be expanded to include other high impact insect groups, beyond repeatedly proving the principle of spatial difference. Our understanding of this relationship is limited to a few species for which this has been directly quantified (e.g. monarch butterfly,^15^, ^18^, ^49^ *Helicoverpa armigera* 46 (both Lepidoptera) and the hoverfly *Episyrphus balteatus* [Diptera] (δ^2^ *H* only)^17^ or meaningfully extrapolated as provided in various dragonfly (Odonata) species).^30^ Reliable knowledge of these ‘calibration’ relationships, including temporal or seasonal fluctuations if appropriate, will enable the generation of spatially explicit statistical methods appropriate to biosecurity and other ento‐forensic applications. Similar ‘complex biological isoscapes’^50^ have been used to forensically distinguish natal origins of other temporally dynamic substrates such as leaf water^51^ and human hair.^52^

Significantly, our reference isotope dataset reveals significant variation accounted for by season as well as by region. Accordingly, the assessment conducted here does not rely on long‐term, regional averages of δ^2^H and δ^18^O derived from rainfall, as is typical in provenance determination studies.^43^ The global^26^, ^53^ and regional (e.g.)^50^, ^54^ isoscapes that exist for these elements can provide powerful spatial reference data in situations where organisms' isotope ratio reflect such averages. Such an approach was not followed here, as the clade of sap‐sucking insects, yet neither the required temporally resolved calibration relationship nor the spatially relevant precipitation data were available. Therefore, instead, we have use the δ^2^H and *δ* ^*18*^O values from the insects themselves with knowledge of the highly correlated climatic relationship of δ^2^H and δ^18^O to indicate the ‘season of origin’. However, a disadvantage to this approach is that there is no probabilistic assignment to the place of natal origin outside of the reference data geographic coverage.

Regarding further technological development, the detection of single insect samples must be assumed for high‐risk exotic pest species interceptions,^55^, ^56^, ^57^, ^58^ as in the current case. The restricted tissue mass available from such samples has hitherto been analytically rate limiting. Furthermore, in the absence of larger specimen numbers, generating multiple markers from these samples is highly desirable, as this enables more robust statistical examination.^31^, ^59^ Both issues are limitations for other high‐priority pests for which this technology is useful, such as fruit fly (Diptera: Tephritidae), spotted wing drosophila (Diptera: Drosophilidae: *Drosophila suzukii*), aphids (Hemiptera: Aphididae) and longhorn beetles (Coleoptera: Cerambycidae). The case presented here has directed additional methodological development and evaluation in our laboratory for small sample δ^2^H and δ^18^O analysis. This includes trials to minimize O background with a molybdenum‐lined reactor,^60^ utilize CO adsorbents and a secondary GC column, conversion of CO to carbon dioxide (CO_2_),^61^ and miniaturization of the thermolysis reactor.^62^, ^63^ Lastly, advances elsewhere have recognized that multiple isotope markers are more informative than single markers for provenance determination.^20^, ^64^ Combinations amongst the climatically linked light element isotope markers of δ^2^H and δ^18^O with the geologically linked, heavy element isotope ratios such as ^87^Sr/^86^Sr, ^207^Pb/^206^Pb and ^208^Pb/^206^Pb are especially useful.^20^ Moreover, the entomological applicability of heavy element isotope ratio for both long‐distance discrimination and near‐scale resolution has been recently revealed.^65^ However, measurements of these heavy element isotope ratios in small single insect samples has been until recently beyond the lower sensitivity of the existing analytical technology. Looking forward, the development of analytical methods for these markers in restricted mass biological samples is possible^66^, ^67^ and this specific application is being considered.^68^ This later endeavour holds the potential to greatly facilitate the wider application and adoption of biogeochemical tracking in several divergent areas such as criminal forensics, ecology and pest management.
